# The Biological Foundations of Sarcopenia: Established and Promising Markers

**DOI:** 10.3389/fmed.2019.00184

**Published:** 2019-08-13

**Authors:** Martina Casati, Andrea Saul Costa, Daniele Capitanio, Luisa Ponzoni, Evelyn Ferri, Simone Agostini, Elisa Lori

**Affiliations:** ^1^Geriatric Unit, Fondazione IRCCS Ca' Granda, Ospedale Maggiore Policlinico, Milan, Italy; ^2^IRCCS Fondazione Don Carlo Gnocchi, Milan, Italy; ^3^Department of Biomedical Sciences for Health, University of Milan, Milan, Italy; ^4^CNR Neuroscience Institute, Milan, Italy; ^5^Department of Biomedical and Clinical Sciences L. Sacco, University of Milan, Milan, Italy

**Keywords:** aging, sarcopenia, biomarkers, neuromuscular junction, SNAP25

## Abstract

Sarcopenia, the progressive loss of muscle mass and strength, is one of the major health issues in older adults, given its high prevalence accompanied by huge clinical and socioeconomic implications. Age-related changes in skeletal muscle can be attributed to mechanisms both directly and indirectly related to muscle homeostasis. Indeed, a wide spectrum of age-related modifications in the organism was shown to play a key role in the pathogenesis of sarcopenia. Not surprisingly, sarcopenia has sometimes been indicated as a syndrome stemming from the aging process, and not as univocal standalone disease. Due to the multidimensionality of sarcopenia, a single biomarker approach is not enough to explain the biology of this condition. The aim of this review is to suggest innovative and promising sarcopenia markers investigating the link between skeletal muscle and brain. Indeed, as a neurological origin of sarcopenia has been hypothesized, a new perspective on sarcopenia biomarkers may focus on the dysfunction of the neuromuscular junctions (NMJs). The core SNARE synaptosomal-associated protein of 25 kDa (SNAP25) accumulates in the plasma membrane of nerve terminals at NMJs and regulates exocytosis at peripheral and central synapses. Interestingly, mice studies have shown that SNAP25 affects the neuromuscular function. SNARE complex and, in particular, SNAP25 may represent a promising pathway to explore the molecular and cellular mechanisms regulating muscular homeostasis and concur at profiling the sarcopenia biological background.

## Aging and Sarcopenia

The global increase of human life expectancy and the rapid aging of the population represent emerging major sociodemographic phenomena.

The aging phenotype is heterogeneous and individuals of the same chronological age may dramatically differ in their health status from each other. In this context, over the past decades, the concept of frailty has been used to define age-related condition characterized by increased vulnerability of physiological systems to stressors, exposing individuals to increased risk of negative outcomes (1).

The physical manifestation of frailty is typically described as dominated by the presence of sarcopenia, one of the most notable changes in body composition being defined by the loss of skeletal muscle mass and strength (2).

The prevalence of sarcopenia in persons aged 60–70 years old is reported to be between 5 and 13%, but it increases to 11–50% in people older than 80. Starting since the age of 40 years, individuals lose 1–2% of muscle per year. At the age of 70 years, 25–30% of skeletal muscle mass is lost, and muscle strength even more pronouncedly is declined by up to 40% (3).

In older persons, the etiology of sarcopenia is multi-factorial. Under normal circumstances, physiologic muscle mass and function are related to a dynamic balance between positive and negative regulators of muscle growth. The central nervous, immune, and endocrine systems coordinate this balance. The sarcopenia onset and progression depend on a combination of mechanisms, typically involved in the aging process, whose combination results in the disruption of the normal physiology of skeletal muscle (4).

Among the age-related changes in body composition occurring at the level of the muscle fiber structure, modifications of its contractile properties and abnormalities of neuromuscular junctions (NMJs) are particularly evident. In particular, sarcopenia shows a muscle fiber distribution with a predominance of type I fibers correlated to an atrophy of type II fibers (5), and an accumulation of adipose tissue both around and between muscle fibers (6). Notably an altered protein metabolism and a dysregulated autophagy leading to muscle atrophy play a role as well in this phenomenon. The picture is even more complex because mechanisms indirectly related to muscle homeostasis also participate in the pathogenesis of sarcopenia (i.e., hormonal status, inflammatory status, insulin resistance, telomere shorting, and oxidative stress).

More recently, a neurological origin of sarcopenia has been postulated (7). Since the integrity of the neurophysiological functions plays a pivotal role for the maintenance of the skeletal muscle in older persons (8), it has been hypothesized that a brain-muscle link could underlie of the sarcopenia development. The assumption that the muscle contractile activity is regulated by the central nervous system (CNS) at the NMJs suggests that the functionality of the NMJs is indispensable for the maintenance of both motor nerve and muscle fibers (9). NMJs have the characteristic structural features of chemical synapses acting as interface between nervous and skeletal muscular system. Age-related alterations in the nervous system play pivotal a role in the musculoskeletal impairment and the decline of the muscle power generation (10). Such age-related remodeling includes loss of motor neurons and transformations of motor units through collateral reinnervation (11), impairment of neuromuscular activation (12), and uncoordinated patterns of intermuscular neural activation (13).

Under these premises, it is evident that sarcopenia is characterized by a peculiar multidimensionality and a consequent heterogeneity. For this reason, the “one-fits-all” approach, where a single process is able to explain the clinical manifestation, may be inadequate for capturing the inner biological foundations of this condition. Thus, a single biomarker is not enough to explain the biology of this complex age-related condition and cannot distinguish sarcopenia from a mere condition of advanced age. Only considering a multidimensional strategy to discover biomarkers of sarcopenia it will be possible to isolate the real fingerprints of this disease.

This review aims to summarize the available literature on sarcopenia biomarkers and to propose innovative and promising ones. Under this perspective, we extended the investigation of the non-muscle-specific sarcopenia biomarkers deepening the analysis of the molecular pathway related to the brain-muscle link.

## Biomarkers of Sarcopenia

In recent years, many studies on sarcopenia have identified numerous biochemical biomarkers. These biomarkers could be divided in “muscle-specific” and “non-muscle-specific.”

### Established Biomarker of Sarcopenia

#### Muscle-Specific Biomarkers

The muscle-specific biomarkers should ideally be able to evaluate both muscle mass and strength loss, the two dimensions of sarcopenia.

Several techniques are used to quantify muscle mass. The measurement of muscle or fat free mass is usually obtained by using imaging assessments as the dual energy X-ray absorptiometry, the computerized tomography, the magnetic resonance imaging, or bioelectrical impedance analysis. However, the current lack of a univocal operational definition and the absence of a clear “gold standard” assessment method make the diagnostic problem still problematic.

Multiple tests of physical performance are available, including the gait speed, the Short Physical Performance Battery (SPPB), the 6-min walk test, and the stair climb power test. Unfortunately, each of them has own characteristics and only captures specific aspects of the muscle functioning, thus concurring at the heterogeneous set of possibilities in the measurement of sarcopenia.

In the last years, a number of muscle-specific biomarkers of sarcopenia have been identified. Recently, several studies have raised doubts about the specificity of these markers in detecting sarcopenia.

Among the “muscle-specific” biochemical markers there are several linked to muscle turnover. Procollagen type III N-terminal peptide (P3NP) is a fragment released by the proteolytic cleavage during collagen synthesis in the muscle. It represents an interesting marker for the analysis of skeletal muscle remodeling. Evidence shows that serum P3NP concentrations reflect the muscle mass (14). However, P3NP levels are altered also in autoimmune diseases as psoriasis, psoriatric arthritis, and rheumatoid arthritis (15).

The peptides deriving from the collagen type VI turnover, as a type VI collagen N-terminal globular domain epitope (IC6) and MMP-generated degradation fragment of collagen 6 (C6M) could be potential markers for the muscle loss.

Collagen type VI has been proposed as a biomarker of muscular tissue damage (16). This protein is important for the preservation of muscle trophism; in fact, genetic defects of this protein are linked to very serious muscular diseases. Nevertheless, these molecules were found to be altered also in serum of cancer patients (17).

The 3-methylhistidine (3MH) results from the methylation of histidine residues of actin and myosin, and induces proteolysis of myofibrils; evidence showed that it could be implicated in the sarcopenia pathophysiology (18). It must be taken into consideration that 3MH is strongly influenced by dietary habits and meat consumption (19).

Skeletal muscle-specific isoform of troponin T (sTnT) may be considerate a biomarker of sarcopenia, since high troponin levels are an expression of muscle wasting. Abreu et al. (20) showed that serum levels of cardiac troponin T (cTnT) decrease in relation to a physical performance improvement, opening new hypotheses about this molecule as marker of muscle damage. It is well-known that cTnT is a biomarker of cardiac dysfunctions as well as diabetes, pulmonary arterial hypertension, and chronic kidney disease (21).

The fact that creatine is related to the skeletal muscle state should also not underestimated. Urinary creatine excretion is correlated with the skeletal muscle mass of total body, and increasing evidences demonstrate that creatine supplementation results in positive effects on muscle strength (22). However, urinary creatine excretion is also altered in other organism dysfunctions as testicular damage (23).

#### Non-muscle-specific Biomarkers

A lot of non-muscle-specific biomarkers were found to be related to sarcopenia pathology. However, these markers are also involved in mechanisms underlying the aging process and could be modified by environmental factors as lifestyle, comorbidity, diseases, and exposure to drugs. In particular:

- *Markers of inflammatory system*. An imbalance between pro- and anti-inflammatory pathways, responsible for a smoldering low-grade inflammation (the so-called “inflamm-aging” phenomenon) characterizes aging (24). In this situation increased levels of pro-inflammatory cytokines and reduced serum level of anti-inflammatory cytokines are observed. Inflamm-aging could contribute to sarcopenia exerting pro-sarcopenic effects through the inhibition of muscle regeneration (25). Indeed, in older adults, up-regulated levels of C-reactive protein, interleukin-6, interleukin-8, tumor necrosis factor-α, interferon-γ, granulocyte-monocyte colony-stimulating factor, and high-temperature requirement serine protease A1 are related to muscle mass, strength and physical function impairment (26). Moreover, high circulating extracellular heat shock protein 72, a mediator of inflammatory system, results in low muscle mass, weak grip strength, and slow walking speed (27).
- *Markers of oxidative damage*. Oxidative stress and the accumulation of reactive oxygen species increase with aging, and are likely to play an active role in triggering sarcopenia. If free radicals are not eliminated by the anti-oxidant agents, they cause severe damage on muscle cells. Evidence shows that serum concentrations of protein carbonyls, well-known markers of oxidative stress, are related with grip strength. Advanced glycation end products (AGEs) are bioactive compounds formed by non-enzymatic glycation of DNA, proteins, and lipids. Elevated serum levels of AGEs are linked to muscle strength impairment (28), however altered expression of AGEs is related also to diabetes (29).
- *Markers of nutritional status*. In older persons the physical performance impairment is associated to anemia with low hemoglobin or low serum albumin and/or selenium (30).
  Leptin seems to affect the skeletal muscle, through the modulation of lipolysis and insulin sensitivity. Considering that the muscle is the major user of glucose, sarcopenia may represent a risk factor for the development of insulin resistance (31). Evidences showed an increase of circulating levels of leptin in sarcopenia due to a reduction of leptin receptors along with a loss of muscle mass.
  In elder people, positive relationships between plasma levels of uric acid and handgrip (32) and between magnesium levels and indexes of muscle performance were observed (33).
  Moreover, the skeletal muscle health is linked to vitamin D. Vitamin D affects muscle cell contractility, proliferation, and differentiation (34). Vitamin D insufficiency results in muscle deficits and vitamin D supplementation has beneficial effects. Further, the identification of vitamin D receptor in skeletal muscle provides evidence for its direct effect on sarcopenia (35).
- *Markers of endocrine system*. Loss of muscle mass is also caused by the reduced capacity for synthesizing proteins and repairing muscle damage which implies a progressive switch from anabolic to catabolic metabolism. It was found that defects in muscle protein homeostasis might be associated to changes in circulating levels of hormones. Studies showed that sex hormones, including testosterone and dehydroepiandrosterone sulfate (DHEAS), whose levels decrease during age, have a role in the sarcopenia onset (36). Testosterone has an anabolic, anti-catabolic, and anti-inflammatory effect on muscle, stimulating cell activation, proliferation, survival, and differentiation, thus playing a role in maintenance of muscle homeostasis (37). DHEAS may affect muscle performance and its age-associated decline is an important determinant of muscle mass and strength loss in older people (38). Sarcopenia is characterized also by a decline of the growth hormone (GH) and the insulin-like growth factor 1 (IGF-1). The actions of GH are mediated by IGF-1, an anabolic hormone, which stimulates muscle growth and regeneration. Giovannini et al. (39) demostrated that IGF-1 administration increases the rate of skeletal muscle functional recovery after injury.
- *Markers related to growth factors*. Myostatin and the growth differentiation factor-15 (GDF-15), members of the transforming growth factor-β (TGF-β) superfamily, are negative regulators of skeletal muscle myogenesis and inhibitors of muscle growth. Interestingly, it was found that older myostatin-null mice show resistance to the sarcopenic phenotype (40) and the use of antibodies against myostatin lead to an improvement of muscle mass and grip strength (41). Follistatin, a myostatin inhibitor, seems to be an interesting tool in evaluating the muscle damage (42). Another sarcopenia biomarker is brain-derived neurotrophic factor (BDNF), which induces the production of growth factors associated with differentiation, plasticity, and neuronal growth. In skeletal muscle, BDNF is involved in the regulation and survival of motoneurons, in the development and differentiation of myoblasts, in the interaction between immune cells and muscle cells (43). However, myostatin and follistatin are altered in osteoarthritis (44, 45), GDF-15 in cardiovascular disease (46), and BDNF in mood disorders (47) and deregulated heart autonomic system (48).
- *Markers of NMJ dysfunction*. In the skeletal muscle, NMJs are implicated in the transduction of the action potentials, and their dysfunction can lead to a gradual alteration of this process. The structural features of NMJs are similar to chemical synapses and many molecules involved in the formation of the NMJs have an essential role in their maintenance (49). Agrin is a heparan sulfate proteoglycan synthesized at the levels of motor neurons, transported along axons and released into the synaptic basal lamina of the NMJs. Here agrin stimulates the assembly of the postsynaptic apparatus, including the clustering of acetylcholine receptors and the stabilization of presynaptic structures. Agrin is fundamental for the formation and stabilization of NMJs (50). The proteolytic cleavage of agrin at the NMJs by neurotrypsin produces a C-terminal 22-kDa fragment (CAF), which is released into the circulation and can be detected in human serum. Elevated serum CAF levels resulting from NMJs disassembly and denervation are associated with sarcopenia (51) and kidney dysfunction (52).

### Promising Biomarker of Sarcopenia

#### Core SNARE Synaptosomal-Associated Protein of 25 kDa (SNAP25)

**Figure 1:**
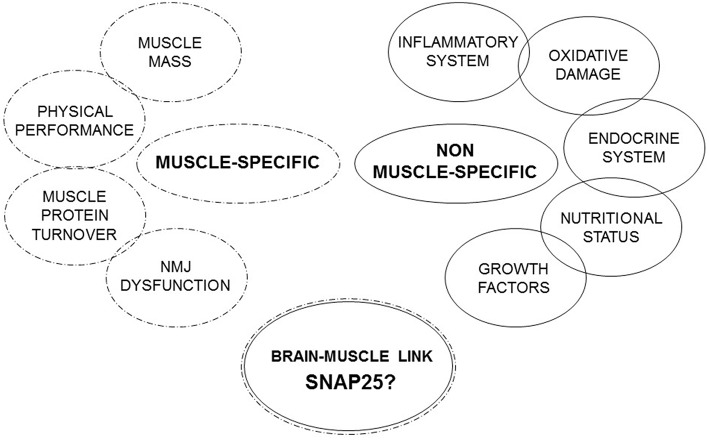
Muscle-specific and non-muscle-specific factors involved in sarcopenia.

Under these premises, a single biomarker approach is not enough to explain the real fingerprints of sarcopenia and no single established sarcopenia biomarker is specific for this condition. Therefore, in this review we propose to focus on molecules that spanning different systems and organs studying the link between brain and muscle (Figure 1).

The core SNARE synaptosomal-associated protein of 25 kDa (SNAP25) could represent a promising pathway. SNAP25 plays a central role in the release of neurotransmitters, inhibiting the voltage-gated calcium channels (VGCC) function and reducing responsiveness to depolarization. SNAP25 accumulates in the plasma membrane of nerve terminals at NMJs, consistent with its SNARE protein role in regulating exocytosis at peripheral and central synapses. In CNS, SNAP25 participates with syntaxin-1 and synaptobrevin/VAMP2 (53) in the regulation of synaptic vesicle exocytosis. SNAP25 has two isoforms, SNAP25a and SNAP25b, resulting from alternative splicing of the exon 5 of the SNAP25 gene, which are differentially expressed during the development. The reduction of this protein levels has been reported to negatively affect synaptic function (54) and to be related even to age-related diseases (i.e., Alzheimer's disease and diabetes) (55, 56). SNAP25 modulates VGCCs, it negatively controls neuronal calcium responsiveness to depolarization through VGCC inhibition (57). Consistently, silencing endogenous SNAP25 in glutamatergic neurons results in increased VGCC activity.

SNAP25 is synthesized in mouse motor nerve endings and the level of SNAP25 mRNA affects neurotransmitter exocytosis (58). Furthermore, neuromuscular transmission is compromised in the presence of a mutation in SNAP25b gene that inhibits synaptic vesicle exocytosis (59). Notably, adult SNAP25+/– mice express low protein levels and are characterized by a decrease of neuromuscular strength (60), suggesting that reduced levels of this protein negatively affect neuromuscular function. These observations seem to be supported by recent data showing that zebrafish embryos model motor impairment, increased motility and neurotransmitter secretion were associated with an over-expression of SNAP25 (61).

## Conclusions and Future Perspectives

Even if sarcopenia and aging share common molecular and cellular mechanisms, a definitive pathophysiological framework of these conditions is still absent. There is, thus, the need of employing a more comprehensive approach to study the age-related changes of body composition leading to sarcopenia. In particular, it is necessary to recognize the inadequacies of previous paradigms, too often investigating sarcopenia as a standalone disease, independently of the aging process.

Under this perspective it is necessary to deepen the study of the brain-muscle link. NMJ seems to be the most innovative and attractive marker of sarcopenia to study. This aspect represents an important conceptual advance, because till now research on sarcopenia has mainly focused on the study of muscular factors and less on the possible role of neurological mechanisms in its onset and development. Therefore, the SNARE complex and, in particular SNAP25, may represent a promising pathway to explore the molecular and cellular mechanisms regulating muscular homeostasis and NMJs. In conclusion, the proposed SNAP25 pathway could represent a new piece enriching the panel of the established biomarkers of sarcopenia (Figure 1).

## Author Contributions

All authors listed have made a substantial, direct and intellectual contribution to the work, and approved it for publication.

### Conflict of Interest Statement

The authors declare that the research was conducted in the absence of any commercial or financial relationships that could be construed as a potential conflict of interest.
